# Monitoring of *n*-butanol vapors biofiltration process using an electronic nose combined with calibration models

**DOI:** 10.1007/s00706-018-2243-6

**Published:** 2018-08-10

**Authors:** Bartosz Szulczyński, Piotr Rybarczyk, Jacek Gębicki

**Affiliations:** Department of Chemical and Process Engineering, Faculty of Chemistry, Gdańsk University of Technology, Gdańsk, Poland

**Keywords:** Odorous substances, Sensors, Mathematical modeling, Alcohols

## Abstract

**Abstract:**

Malodorous odors, by definition, are unpleasant, irritating smells being a mixture of volatile chemical compounds that can be sensed at low concentrations. Due to the increasing problem of odor nuisance associated with odor sensations, and thus the need to remove them from the air, deodorization techniques are commonly used. Biofiltration is one of the methods of reducing odorants in the air stream. In the paper, the possibility of using an electronic nose as an alternative method to gas chromatography for the online monitoring and evaluation of efficiency of the *n*-butanol vapors biofiltration process in a transient state was investigated. Three calibration models were used in the research, i.e., multiple linear regression, principal component regression, and partial least-square regression. The obtained results were compared with the theoretical values.

**Graphical abstract:**

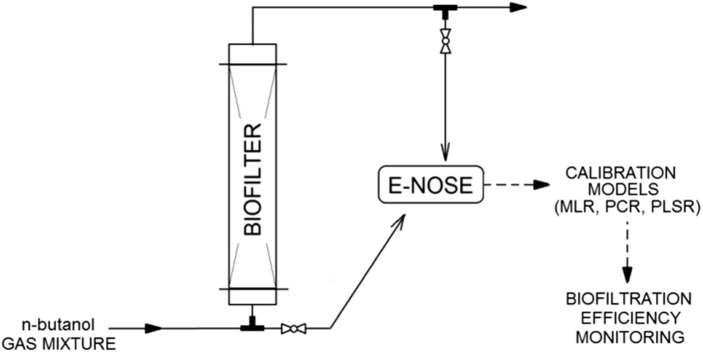

## Introduction

Among various methods of controlling volatile organic compounds’ (VOCs) emissions, biological techniques seem to be very promising due to low operating costs, possibility to treat gases with low concentration of odorants and very low secondary pollution generation. The use of biological methods for air purification has been known for over 60 years. Devices called biofilters are most commonly used for this purpose [1]. A typical biofilter is a column-type bioreactor packed with a bed made from either natural or synthetic materials. The process of biofiltration consists in the decomposition of contaminants by bacteria or other microorganisms located in the biofilm formed on the filter bed elements. The contaminated gas is humidified prior to entering the biofilter. The mechanism of biofiltration involves the diffusion of pollutants from the gas phase to the biofilm, covering the surface of the packing element. The compounds absorbed in the biofilm undergo biodegradation and the clean gas leaves the biofilter [2, 3].

Elements of a biofilter bed are moisturized with an aqueous phase. Therefore, the biofilm formed on the surface of such elements of bed allows for the absorption of hydrophilic compounds, as it is in the case of *n*-butanol. *n*-Butanol belongs to the group of VOCs, concentrations of which need to be controlled in the environment. Emissions of *n*-butanol are identified, i.e., from processes of thermal regeneration of adsorbents, from wastewater treatment plants, and waste disposal plants, coal mines, and other industrial activities [4, 5]. *n*-Butanol is characterized by harsh and alcoholic-like odor type [6], and it is irritating and hazardous to human health when inhaled. The effectiveness of the removal of such kind of compounds in the biofilters depends mainly on the rate of their biodegradation by microorganisms inhabiting the biofilm. This is so because the process limitation with respect to mass transfer of the hydrophilic compounds from the gas to liquid phase is negligibly small [7]. The most crucial parameters affecting the biofiltration efficiency include the type and porosity of the bed, the pressure drop through the bed, pH value, gas flow rate, temperature, and concentration of pollutants in the gas phase [8].

Mathematical modeling is a useful tool for the prediction of a biofilter performance. Biofiltration models usually follow a transfer-reaction scheme [9]. Such an approach requires the knowledge of physical (i.e., gas–liquid equilibrium and transfer coefficients) as well as biological (kinetics and efficiency of biodegradation) parameters. One of the first and the most popular models describing biofiltration was proposed by Ottengraf [10, 11]. According to [10], the Ottengraf model is based on the following assumptions: (1) the mass transfer resistance in the gas phase is negligible comparing to the mass transfer resistance in the liquid phase; (2) the biofilm thickness is much smaller than the diameter of a bed element; (3) the biofilm may be treated as a planar surface; (4) substrate transport through the biofilm occurs by diffusion; (5) the interface between the gas and the liquid phases is in the equilibrium; (6) biodegradation may be described by the Michaelis–Menten kinetics or by the Monod equation; (7) constant kinetic constants may be used due to the net growth of biomass in the biofilm which is controlled to be zero; (8) the biomass is uniformly distributed in the biofilter; (9) the biofilter is treated as a plug flow reactor. The Ottengraf model, proposed in 1983, is a rather simple model predicting the performance of the biofilter; however, it is still treated as a reference for other models and their modifications [12]. When modifying or developing a new model for the description of biofiltration, following problems must be taken into consideration: (1) an influence of unstable conditions during the biofiltration, (2) the possibility of interactions between the gas-mixture components, and (3) the identification of biodegradation inhibitors.

**Fig. 1:**
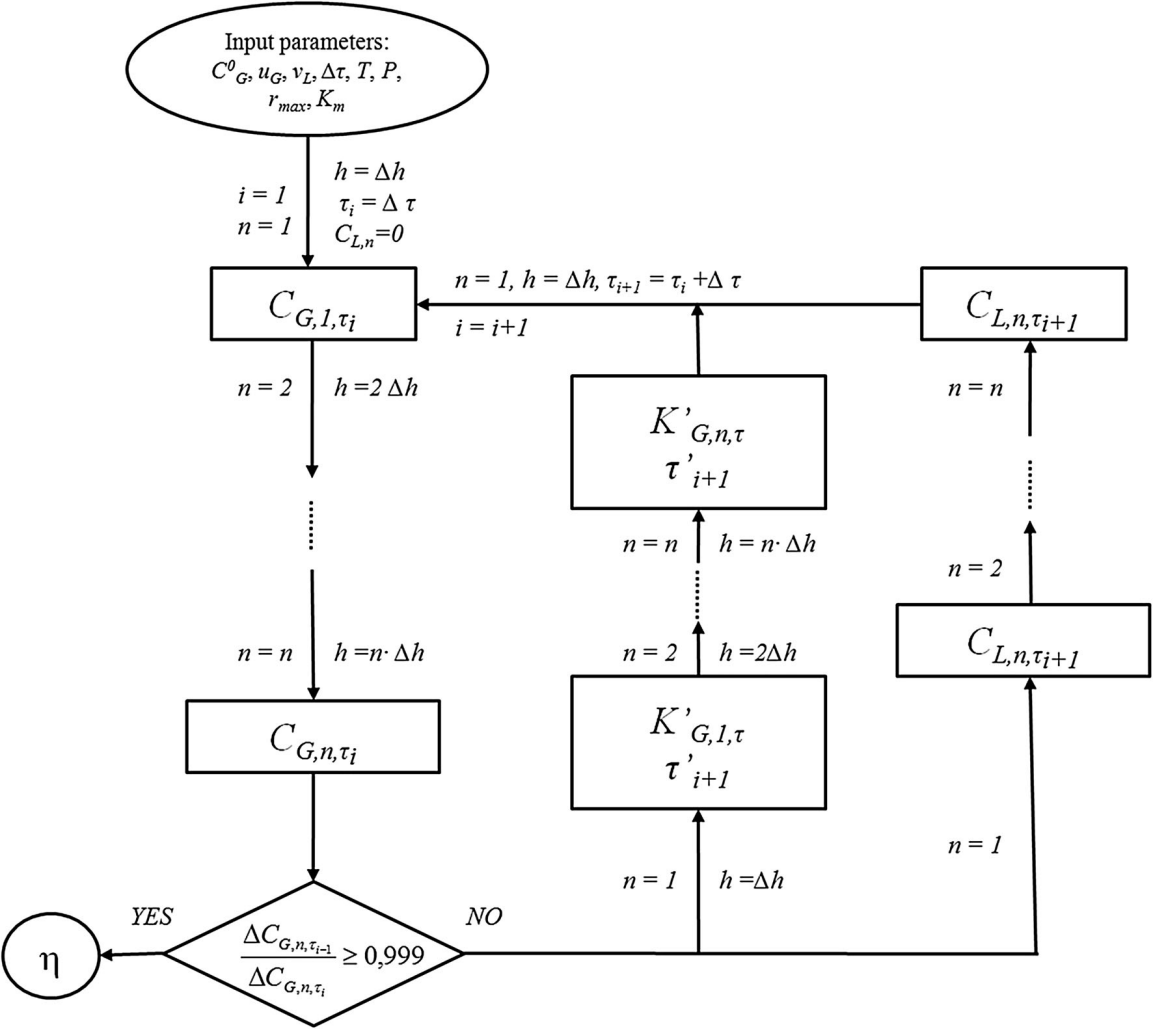
Algorithm for calculating the concentration of *n*-butanol (*C*_G_) in the outlet stream at the height *h* after the process duration time *τ*_*i*_. *u*_*G*_ gas velocity, *υ*_*L*_ liquid viscosity, ∆*τ* time interval, *T* temperature, *P* pressure, *r*_*max*_ maximum biodegradation rate, *K*_*m*_ Michaelis–Menten constant, *n* number of height intervals, *i* iteration step, *K*_*G*_ mass transfer coefficient, *η* biofiltration efficiency

Until the steady-state process conditions are established, the system is subjected to both first- and zero-order reactions. In addition, for a given moment of the process duration at two different horizontal cross-sections of the biofilter, the occurring reactions proceed with different rates and may follow the kinetics of different order. Therefore, it is impossible to determine the order of reactions in the initial period of the biofiltration process, because the process occurs in unsteady-state conditions. For this reason, the model proposed by Świsłowski [13] was used in this paper to predict the theoretical values of the time-dependent distribution pattern of n-butanol concentrations in the biofilter outlet gas stream. The calculation algorithm is shown schematically in Fig. 1.

The determination of concentration of a compound at the inlet and outlet of a biofilter allows to calculate the efficiency of the process:

1 $$ \eta = 1 - \frac{{C_{\text{outlet}} }}{{C_{\text{inlet}} }}, $$

where *η* is the biofiltration efficiency; *C*_inlet/outlet_ is the concentration of the odorous compound in the inlet and outlet streams.

**Fig. 2:**
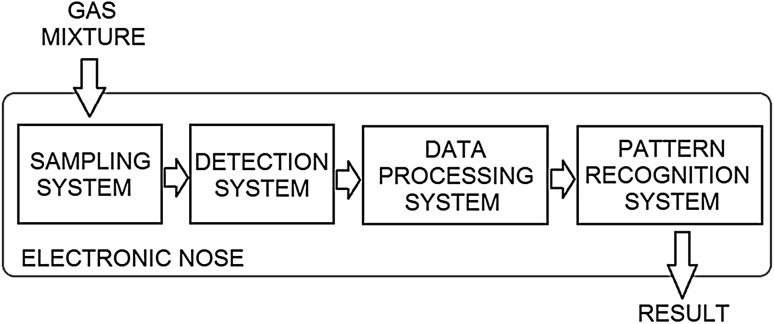
Scheme of an electronic nose system

The most popular methods used for the evaluation of the biofiltration efficiency are chromatographic methods, i.e., gas chromatography coupled with mass spectrometer or flame ionization detector. However, due to the high operating costs, the need to provide high purity gas or vacuum, these techniques are mainly exploited for laboratory tests and are rarely used for the monitoring of industrial biofiltration processes in the online mode. In recent years, there has been a significant increase in the interest in using electronic noses for quantitative and qualitative analyses for environmental monitoring [14, 15]. Due to the short time and low cost of a single analysis, electronic noses have become an alternative to gas chromatography. Electronic noses (e-noses) are devices that are supposed to mimic the human sense of smell and are used in many areas of human activity [16–20]. Such devices consist of four main components, as given in Fig. 2.

The applied sampling system eliminates possible undesirable factors affecting the sensor response, and provides stable and reproducible measurement conditions (temperature, humidity, and gas flow velocity). Detection system is a set of sensors located in the measurement chamber. The most commonly used sensor types are commercially available sensors for the detection of volatile organic compounds, e.g., metal oxide sensors (MOS) [21]. Such sensors exhibit different selectivity and sensitivity, but, when coupled in a sensor matrix, produce a characteristic chemical image of the gas mixture (“fingerprint”). This image is transferred to the data collection system, which is responsible for the digital signal processing. The last but the most important element of the electronic nose system is a pattern recognition system, which assigns the received set of signals to one of the pattern classes or predicts the concentrations using the appropriate mathematical calibration model. The most commonly used models include multiple linear regression (MLR), principal components regression (PCR), and partial least-squares regression (PLSR). Construction of the above models utilizes a set of explaining variables (signals from the sensors comprising an electronic nose) and a set of dependent variables (values of substance concentrations in a gas mixture). A task for the calibration method is to develop a model allowing for the quantitative evaluation of a particular property or the properties based on the set of explaining variables. These methods find successful applications for the monitoring of the changes of concentrations of odorous compounds during biofiltration or sewage treatment [22–25].

Multiple linear regression generates a linear relationship between a single dependent variable (*y*) and a set of several explanatory variables (*x*):

2 $$ y = a_{0} + a_{1} \cdot x_{1} + a_{2} \cdot x_{2} + \cdots + a_{n} \cdot x_{n} . $$

The model can be used when the number of variables is smaller than the number of samples and when these variables are poorly correlated. In other cases, it is impossible to determine the regression coefficients (*a*), which are obtained by the least-squares method [26].

Principal component regression assumes reducing the number of explanatory variables by selecting few first principal components (PCs) in the place of the primary variables. The guiding idea of this method is to formulate a relationship between PCs and the expected property of the sample. The method may be applied in two stages and the first stage allows for the determination of the principal components using the principal component analysis (PCA) method. It allows to obtain uncorrelated matrix of variables. The second stage assumes the development of the MLR model with the use of principal components as variables:

3 $$ y = a_{0} + a_{1} \cdot {\text{PC}}_{1} + a_{2} \cdot {\text{PC}}_{2} + \cdots + a_{n} \cdot {\text{PC}}_{n} . $$

The number of principal components (*n*) most often needs to be selected experimentally. The proper choice is of a great importance for the prognostic capabilities of the model. In the case when too few main components, the calibration model contains insufficient information necessary for the correct forecasting. However, if the number of main factors is excessive, unwanted information such as a noise or experimental errors is introduced to the model.

The partial least-squares regression is the most commonly used method for the development of multidimensional calibration models. The task of the PLS method, like PCR, is the design of the model based on the mutually orthogonal factors. In the PLS method, such factors are created in different way than in the PCR method. In the PCR method, factors are the principal components created on the basis of the variables matrix only, while, in the PLS method, the relationship between variables and dependent variables matrices is taken into account. Each factor in the PLS method explains the maximum covariance between factors for the variables and factors for the dependent variables. Covariance combines a high variance of the variables matrix with a high correlation of the property of interest [27–29].

In the present paper, an electronic nose prototype combined with three types of calibration models (MLR, PCR, and PLSR) is used for the monitoring of *n*-butanol vapors biofiltration process. Research results are compared with theoretical values.

## Results and discussion

**Fig. 3:**
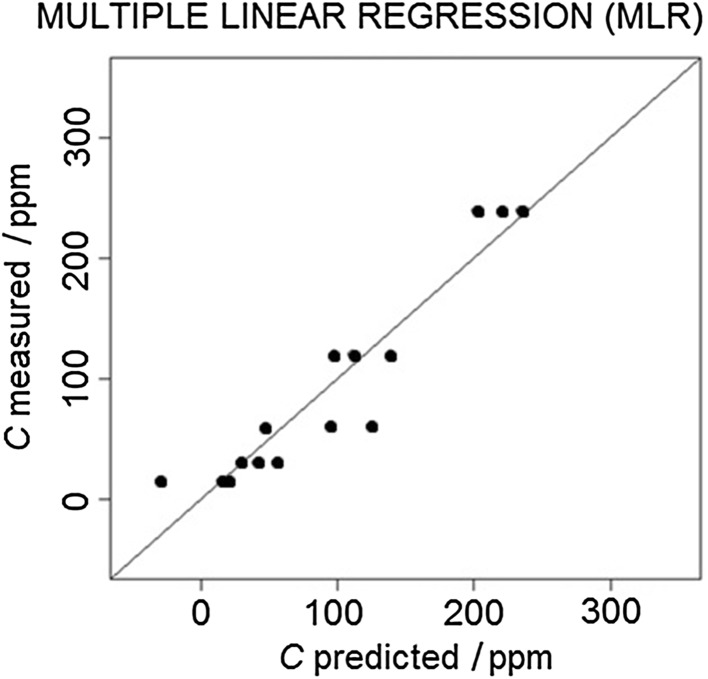
Multiple linear regression model correlation plot

**Fig. 4:**
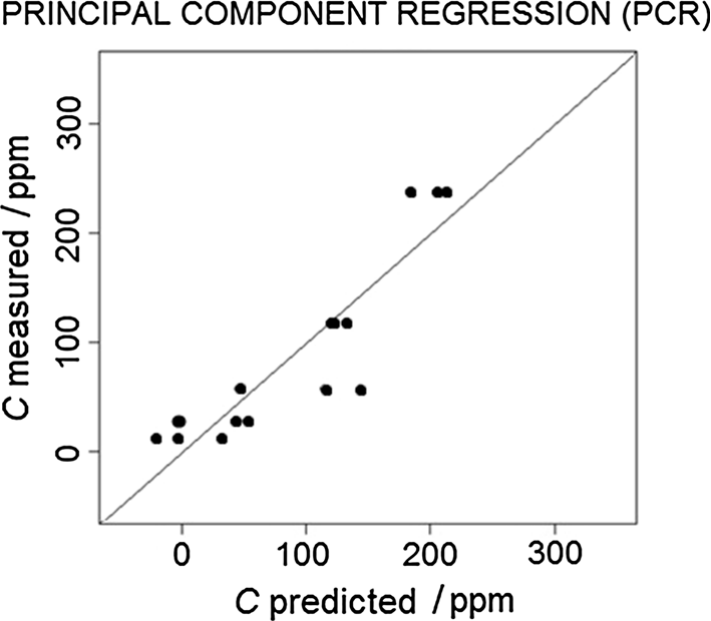
Principal component regression model correlation plot

**Fig. 5:**
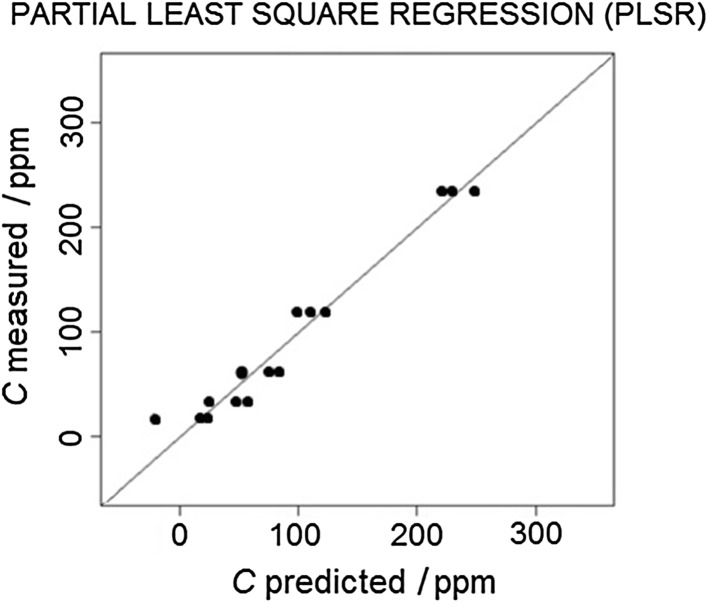
Partial least-square regression model correlation plot

For the data calibration, three models were developed, i.e., MLR, PCR, and PLSR. As a visual method of assessing the fit of the models to the experimental data, the correlation plots were prepared (Figs. 3, 4, 5).

The root-mean-square error of prediction (RMSEP) was chosen as the numerical tool for the selection of the optimal model. The values of RMSEP for the developed models are given in Table 1.

**Table 1:** Root-mean-square error of prediction (RMSEP) values for the developed models

Model	RMSEP/ppm
Multiple linear regression (MLR)	7.5
Principal component regression (PCR)	12.8
Partial least-square regression (PLSR)	5.4

The results of the conducted research indicate that the most suitable calibration model for the determination of the *n*-butanol concentration is the PLS model (i.e., characterized by the lowest value of RMSEP = 5.4), and the least suitable model is PCR with the RMSEP value of 12.8. The obtained results show that, with a relatively small number of training data, PLSR models perform better than in the case of PCR methods. The multiple linear regression method, in terms of the value of mean square error, is placed between PLSR and PCR. It can be concluded that its estimation of the explained variable is relatively good (RMSEP = 7.5), taking into account a small number of degrees of freedom of the model.

**Fig. 6:**
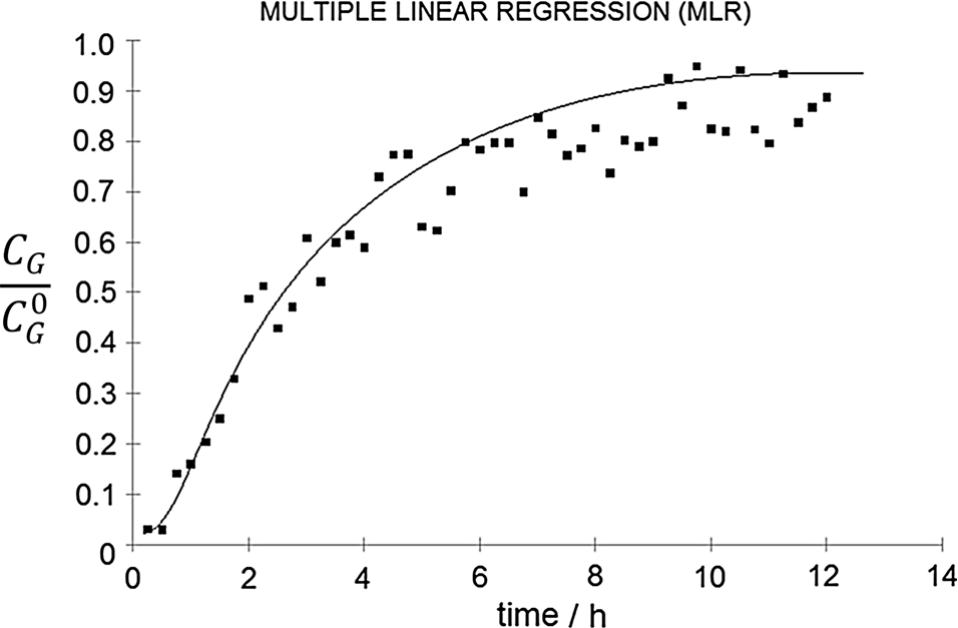
Ratio of outlet-to-inlet *n*-butanol concentration as a function of biofiltration time determined using MLR model (points) compared with the theoretical values (line)

**Fig. 7:**
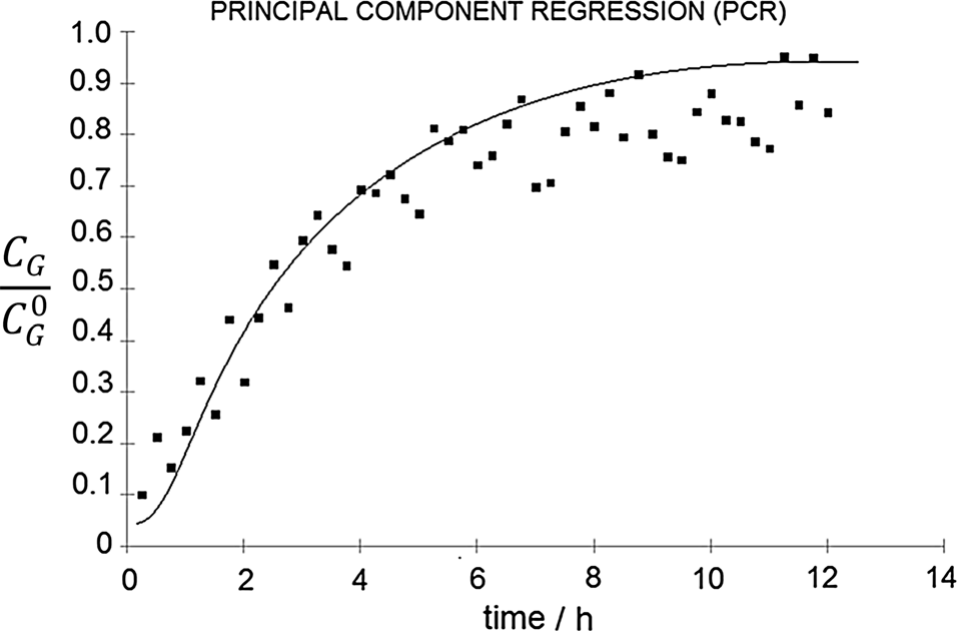
Ratio of outlet-to-inlet *n*-butanol concentration as a function of biofiltration time determined using PCR model (points) compared with the theoretical values (line)

**Fig. 8:**
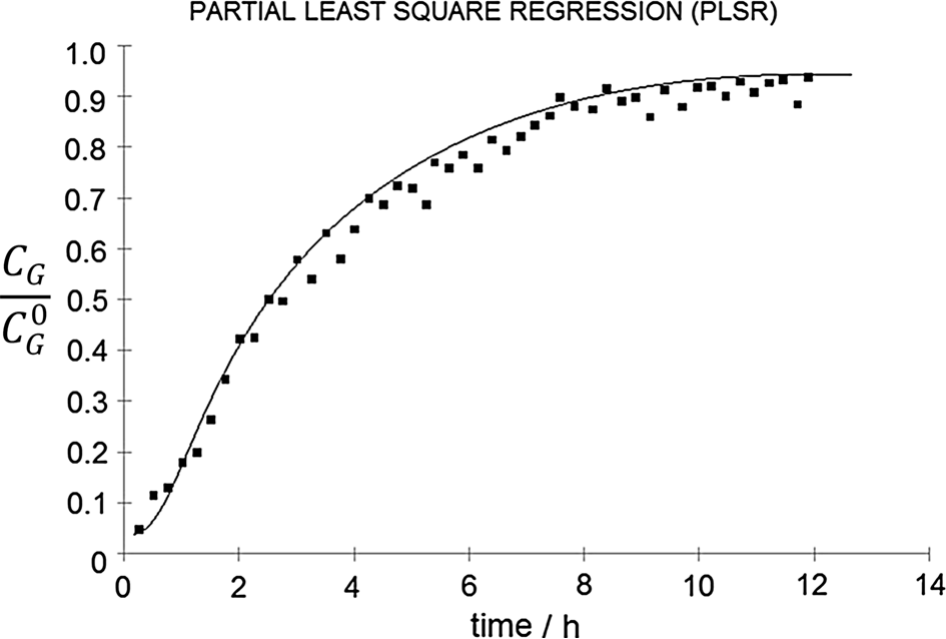
Ratio of outlet-to-inlet *n*-butanol concentration as a function of biofiltration time determined using PLSR model (points) compared with the theoretical values (line)

Good predictive capabilities of the developed models allowed to investigate the possibility of using the e-nose as a tool for monitoring and assessing the effectiveness of the *n*-butanol biofiltration process in unsteady-state conditions. In this part of the work, also the three models were applied (i.e., MLR, PCR, and PLSR). Using the electronic nose, the concentrations of *n*-butanol vapors at the inlet and outlet of the biofilter were determined for the process duration time from the start-up until reaching the stable process conditions. The obtained results (the ratio of the outlet-to-inlet concentrations) as a function of time are shown in Figs. 6, 7, and 8. Presented figures contain also the theoretical values of process efficiency calculated on the basis of the biofiltration model described in Fig. 1.

Very good correlation between the experimental and theoretical results was obtained. The PLSR model presents the best fit to the theoretical curve. For the process duration time longer than about 5–6 h, the MLR and PCR models predicted lower values for the outlet concentration of *n*-butanol (*C*_G_) than the theoretical values. The largest discrepancies were observed for the PCR model which may be due to the presence of water vapor released from the filter bed, which was not present in the calibration samples. This problem has not been observed for the PLSR mode, which is the least susceptible to changes in the sample matrix composition among the investigated models.

## Conclusions

It was found that the constructed electronic nose prototype together with the developed mathematical calibration models (MLR, PCR, and PLSR) can be successfully applied for the monitoring and efficiency assessment of the *n*-butanol vapors biofiltration process. The proposed models were characterized by a high compliance with the theoretical values. The best fit quality was obtained for the PLSR model.

The obtained results confirm that the use of electronic noses as an alternative method to gas chromatography for the online monitoring of the biofiltration process is possible. Due to the low cost of the prototype and short time as well as low cost of a single analysis, the use of e-noses seems to be justified and purposeful for such applications. The possibility of obtaining the results in the online mode highlights the perspective of using e-noses as measuring elements in the automation systems for control and management of air biofiltration processes.

## Experimental

**Fig. 9:**
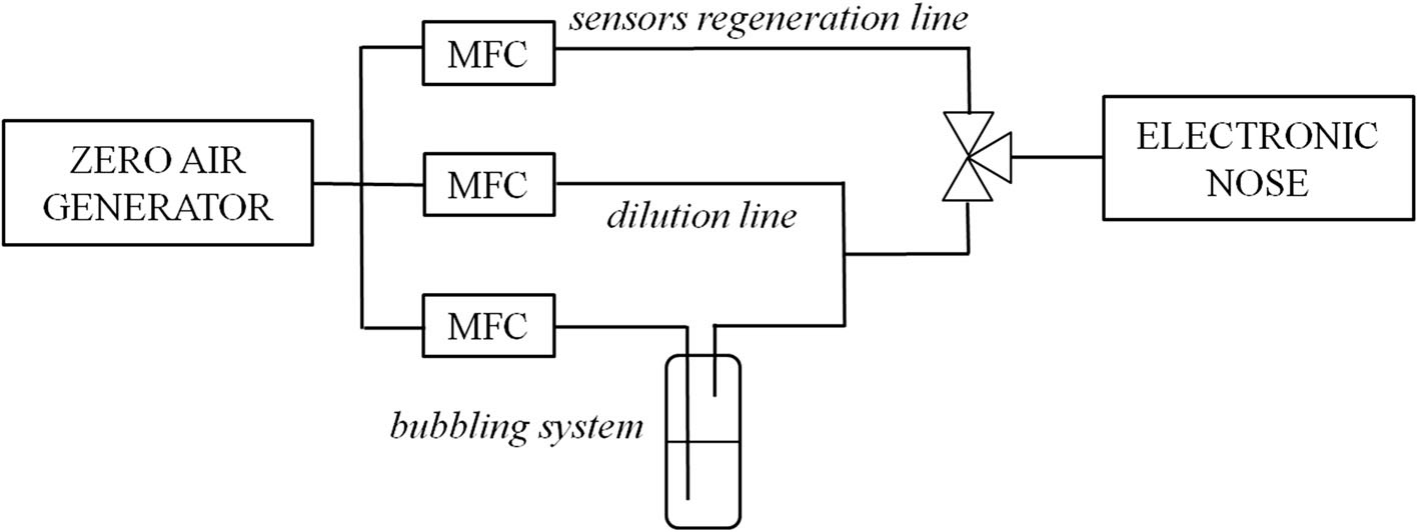
Gas-mixture generation system

**Fig. 10:**
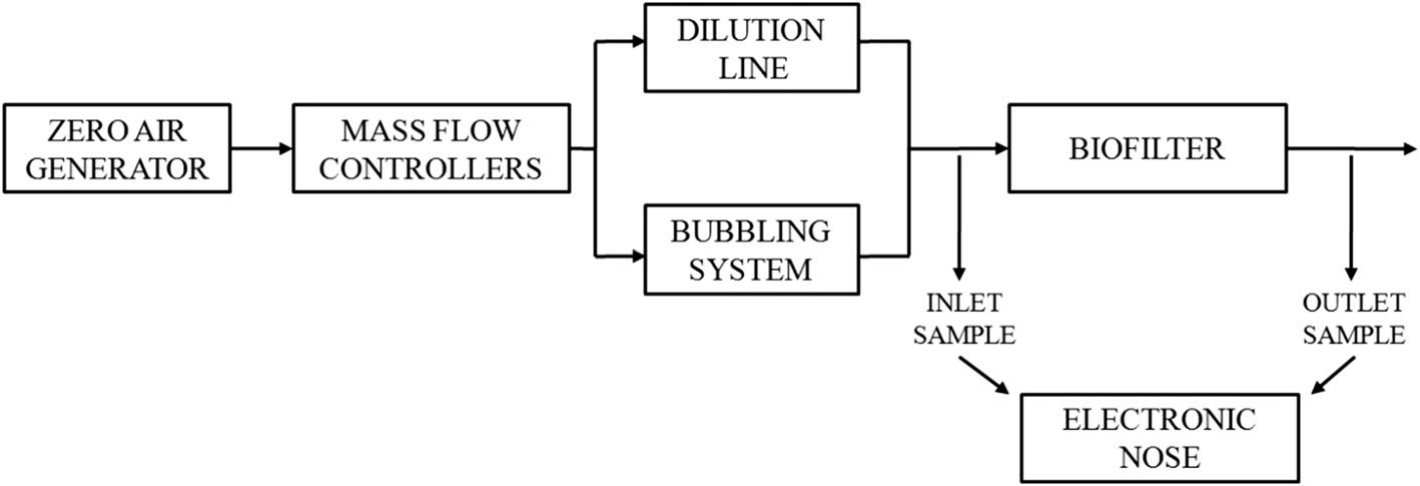
Biofiltration system

Two research systems were used in the investigations: the first was used to develop calibration models for the electronic nose (Fig. 9) and the second system was used for the evaluation of a biofilter performance (Fig. 10).

Mixtures of air and *n*-butanol vapors were generated using the bubbling phenomenon. Purified and dried air was passed through a vial containing *n*-butanol (Sigma-Aldrich). The formed mixture was diluted with a zero air stream to achieve the desired concentration of *n*-butanol in the mixture. The concentration was determined by measuring the weight loss of *n*-butanol in a vial according to the relationship:

4 $$ C = \frac{\Delta m}{V \cdot t}, $$

where *C* is the concentration, Δ*m* is the vial mass change, *V* is the air flow rate, and *t* is the time.

The air flow rates through the vial and the diluting air were controlled by mass flow controllers. Switching the three-way valve, the generated mixture was directed to the electronic nose working in the stop-flow mode (flow time: 30 s; stop time: 15 s). Then, the valve was switched again and zero air was introduced to the e-nose for sensors regeneration (for the time interval equal to 10 min). Measurements were made for five concentrations of *n*-butanol in the mixture: 15, 30, 60, 120, and 240 ppm. Each measurement was repeated three times.

The biofiltration of *n*-butanol vapors was carried out using the system shown in Fig. 10.

A mixture of air and *n*-butanol with a concentration of 240 ppm, generated as described above, was fed to the biofilter. The volumetric flow rate of the mixture through the packed bed was 1.2 dm^3^ min^−1^. Column biofilter was used during the investigations (outside diameter/inside diameter: 0.05/0.04 m, *h* = 1 m). Pine bark elements with an average size of 4–7 mm were used a biofilter packing material. Before starting the process, the bed was sprinkled with an aqueous solution of mineral salts. After the process start-up (process duration time: 12 h), every 15 min, the gas samples were collected and analyzed using the electronic nose prototype, constructed in the Department of Chemical and Process Engineering, Faculty of Chemistry, Gdańsk University of Technology. The device worked with eight TGS metal oxide sensors manufactured by Figaro Inc: TGS 2104, TGS 2106, TGS 2180, TGS 2201, TGS 2600, TGS 2602, TGS 2603, and TGS 2611. The collected sensor signals were saved on the computer using the Simex SIAi-8 analog-to-digital converter. The electronic nose operated in a stop-flow mode. The sample flow time through the chamber was 30 s, while the stop time was 15 s. For the calculations of a sensor signal (*S*), the quotient form was chosen:

5 $$ S = \frac{\Delta S}{{S_{0} }} = \frac{{S_{ \hbox{max} } - S_{0} }}{{S_{0} }}, $$

where *S*_max_ is the maximal value of the signal and *S*_0_ is the sensor baseline value. Data analysis and other calculations were performed using RStudio Desktop (v. 1.0.143) software.
